# Development of a definition for Rapid Progression (RP) of renal function in HIV-positive persons: the D:A:D study

**DOI:** 10.1186/1471-2369-15-51

**Published:** 2014-03-25

**Authors:** David A Kamara, Lene Ryom, Michael Ross, Ole Kirk, Peter Reiss, Philippe Morlat, Olivier Moranne, Christoph A Fux, Amanda Mocroft, Caroline Sabin, Jens D Lundgren, Colette J Smith

**Affiliations:** 1 Research Dept. of Infection and Population Health, University College London, London, United Kingdom; 2 Department of Infectious Diseases and Rheumatology, Rigshospitalet, University of Copenhagen, CHIP, Section 2100, Finsencentret, Copenhagen, Denmark; 3 Division of Nephrology, Mount Sinai School of Medicine, New York, USA; 4 Academic Medical Center, Div. of Infectious Diseases and Dept. of Global Health, University of Amsterdam, Amsterdam, The Netherlands; 5 Service de médecine interne et maladies infectieuses, Hôpital Saint-André, CHU de Bordeaux, France; 6 Nephrology department, Public Health department, CHU Nice, France; 7 Clinic for Infectious Diseases and Hospital Hygiene, Kantonsspital Aarau, Switzerland; 8 Research Department of Infection and Population Health, UCL, Royal Free Campus,Rowland Hill Street, London NW3 2PF, UK; 9 Department of Infectious Diseases and Rheumatology, Rigshospitalet, University of Copenhagen, CHIP, Section 2100, Finsencentret, Blegdamsvej 9 2100, Copenhagen Ø, Denmark

**Keywords:** HIV, Kidney disease, Rapid progression, Estimated glomerular filtration rate, Chronic kidney disease

## Abstract

**Background:**

No consensus exists on how to define abnormally rapid deterioration in renal function (Rapid Progression, RP). We developed an operational definition of RP in HIV-positive persons with baseline estimated glomerular filtration rate (eGFR) >90 ml/min/1.73 m^2^ (using Cockcroft Gault) in the Data Collection on Adverse Events of Anti-HIV Drugs (D:A:D) study from 2004 to 2011.

**Methods:**

Two definitions were evaluated; ***RP definition A:*** An average eGFR decline (slope) ≥5 ml/min/1.73 m^2^/year over four years of follow-up with ≥3 eGFR measurements/year, last eGFR <90 ml/min/1.73 m^2^ and an absolute decline ≥5 ml/min/1.73 m^2^/year in two consecutive years. ***RP definition B:*** An absolute annual decline ≥5 ml/min/1.73 m^2^/year in each year and last eGFR <90 ml/min/1.73 m^2^. Sensitivity analyses were performed considering two and three years’ follow-up. The percentage with and without RP who went on to subsequently develop incident chronic kidney disease (CKD; 2 consecutive eGFRs <60 ml/min/1.73 m^2^ and 3 months apart) was calculated.

**Results:**

22,603 individuals had baseline eGFR ≥90 ml/min/1.73 m^2^. 108/3655 (3.0%) individuals with ≥4 years’ follow-up and ≥3 measurements/year experienced RP under definition A; similar proportions were observed when considering follow-up periods of three (n=195/6375; 3.1%) and two years (n=355/10756; 3.3%). In contrast under RP definition B, greater proportions experienced RP when considering two years (n=476/10756; 4.4%) instead of three (n=48/6375; 0.8%) or four (n=15/3655; 0.4%) years’ follow-up. For RP definition A, 13 (12%) individuals who experienced RP progressed to CKD, and only (21) 0.6% of those without RP progressed to CKD (sensitivity 38.2% and specificity 97.4%); whereas for RP definition B, fewer RP individuals progressed to CKD.

**Conclusions:**

Our results suggest using three years’ follow-up and at least two eGFR measurements per year is most appropriate for a RP definition, as it allows inclusion of a reasonable number of individuals and is associated with the known risk factors. The definition does not necessarily identify all those that progress to incident CKD, however, it can be used alongside other renal measurements to early identify and assess those at risk of developing CKD. Future analyses will use this definition to identify other risk factors for RP, including the role of antiretrovirals.

## Background

Whilst most HIV-positive individuals have a relatively stable renal function over time, some experience a rapid deterioration in their estimated glomerular filtration rate (eGFR) [1-5]. It is important to identify and assess such individuals and potential risk factors, especially those associated with known risk factors for renal insufficiency. These patients are usually difficult to identify, because as soon as their eGFR decreases, but still remains within a clinically acceptable range, most physicians will switch to non-nephrotoxic antiretroviral drugs [2]. Thus, developing a validated definition of an eGFR slope could guide future studies.

Studying those with initially normal renal function (defined by the Kidney Disease Improving Global Outcomes [KDIGO] as eGFR ≥90 ml/min/1.73 m^2^[6]) who experience a rapid decline in their renal function is important, as it could potentially detect kidney impairment at an early stage, before manifest kidney disease has occurred [7,8]. A study from the Modification of Diet in Renal Disease (MDRD) study group, to determine baseline factors that predict the decline in GFR, made a case for rapid decline in renal function to be further investigated. They reported that the mean rate of GFR decline was not significantly related to the baseline GFR, and this suggests an approximately linear mean eGFR decline as renal disease progresses [9]. Rapid progression (RP), an abnormally rapid deterioration in renal function, is a term commonly used in the renal literature. However, unlike CKD (typically defined as two consecutive eGFRs ≤60 ml/min/1.73 m^2^ measured more than 3 months apart), it is without a standardised definition. It is commonly accepted that a normal age-related eGFR decline in the general population (typically >50 years) is approximately 1 ml/min/1.73 m^2^/year [10-12]. Therefore, any definition of RP should consider larger changes, and ≥3 ml/min/1.73 m^2^ has been commonly used [13-18]. However, it is unclear whether a decline of this magnitude is specific enough to accurately identify those at greatest risk of going on to develop CKD. Recent KDIGO guidelines suggest that, for those with evidence of CKD, RP should be defined as a sustained decline of >5 ml/min/1.73 m^2^ per year [12], although no definition is offered for those with normal baseline renal function. Other definitions of RP have also been used, including a >50% decrease in baseline eGFR value [19].

The length of time over which an eGFR decline should be sustained to qualify as RP is also unclear [18]. Furthermore, individuals can have differing numbers of eGFR measurements recorded over follow-up. It remains unclear how these multiple measurements have been accounted for; whether an average annual change (i.e. the averaged slope across all available eGFR measurements for an individual, or the average of several yearly changes) or an absolute change over a time period (i.e. the difference between first and last eGFR measurements) has been calculated. This variation in RP definitions limits the ability to make cross-study comparisons.

HIV-positive persons are at increased risk of renal dysfunction compared to the HIV negative population [1-5], potentially due to high prevalence of traditional renal risk factors [20,21], toxicity from specific antiretroviral drugs [2] and HIV infection itself [5]. As HIV-positive individuals in resource rich settings typically undergo regular renal function testing in accordance with standard screening guidelines, a standardised definition of RP for this population could be beneficial. Therefore, we developed an operational definition for RP in HIV-positive individuals by evaluating the associations between these definitions and traditional renal risk factors. Finally, we investigated the ability of the different definitions to predict future incident CKD.

## Methods

### Study population

The D:A:D study is a large observational multi-cohort collaboration of HIV- positive individuals from 11 cohorts in Europe, USA and Australia [22]. All participating cohorts have obtained ethical approval and, if appropriate, informed consent, as required by national guidelines and regulations. Of particular interest to this present analysis, all creatinine measurements on study participants taken as part of routine care from the date of D:A:D study entry onwards were collected. Data were available from nine contributing cohorts. Only measurements taken after 1st January 2004, the date from which monitoring of creatinine levels was routine across these nine cohorts, were included in this analysis.

### EGFR calculation

As some of the cohorts in the D:A:D study are prohibited from collecting data on participants’ ethnicity, the Cockroft-Gault (CG) formula was used [23-25], standardized for body surface area (BSA), as has been done in a previous D:A:D analysis [2]. The closest weight measurement within 12 months of the date of the creatinine measurement was used, alongside height and current age. Where more than one serum creatinine value was measured within 28 days, the median value and mean date across this time period were used.

### Included study populations

We identified 22,603 D:A:D study participants with at least three eGFR measurements after 1 January 2004 whose first (baseline) eGFR was ≥90 ml/min/1.73 m^2^. We then assessed the proportion of this initial population that would be eligible to be included in a study of RP, depending on the individuals’ length of available follow-up (two, three or four years) and number of measurements per year (≥2 or ≥3) required.

### RP definition

**Figure 1:**
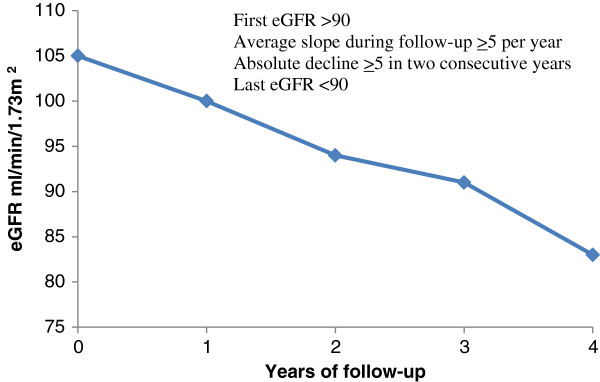
RP definition A.

We next developed two definitions of RP (definition A and definition B). We first considered the study population with at least four years of follow-up, and at least three eGFR measurements in each year. We decided that a relatively large annual decline in eGFR ≥5 ml/min/1.73 m^2^ per year would constitute RP. This was to avoid ‘noise’ introduced by natural eGFR fluctuations, based on advice from nephrologists and in line with the recent KDIGO guidelines [12]. We hypothesised that a RP definition that only considered the difference between the first and last eGFR value (the “absolute decline”) may be too susceptible to random eGFR variation. However, a definition that only considers the modelled decline (i.e. fitting a linear slope to all eGFR measurements during follow-up to estimate the average annual eGFR change) may also not fully capture RP, as negative eGFR changes might be compensated by subsequent positive changes, and so our definition captured both elements. In addition, as the clinical implications of RP within normal eGFR ranges are unknown, we restricted our definition to include only those that progressed to an eGFR value below <90 ml/min/1.73 m^2^. This led to our primary definition of RP (Figure 1) as defined below.

### RP: definition a (RPA; Figure 1)

Individuals were considered to have experienced RP over the four year period if they met all of the following three conditions:

1. Average annual eGFR decline ≥5 ml/min/1.73 m^2^ over four year follow-up period.

2. Absolute eGFR decline ≥5 ml/min/1.73 m^2^ in two consecutive years (defined using the first and last measurements within each year).

3. eGFR <90 ml/min/1.73 m^2^ at the end of the follow-up period.

### Secondary definition for RP: definition B (RPB; Figure 2)

**Figure 2:**
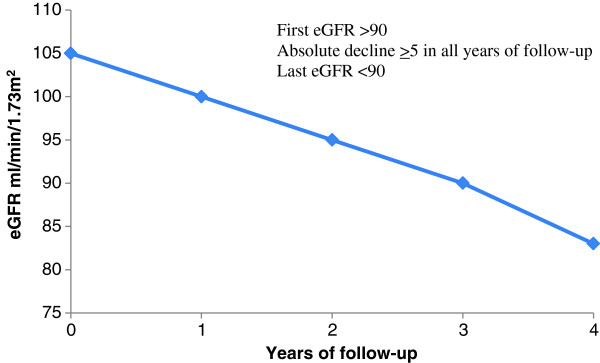
RP definition B.

Individuals were considered to have experienced RP under the more restrictive definition B (Figure 2) if the following criteria were met:

1. Absolute decline ≥5 ml/min/1.73 m^2^/year in *each year* of follow-up (defined on the basis of the first and last measurements within each year).

2. eGFR <90 ml/min/1.73 m^2^ at the end of the follow-up period.

### Alternative RP definitions

The analyses were repeated using different follow-up periods (three and two years instead of four years) and restriction on the number of measurements per year required to be eligible for inclusion in the analysis (≥2 measurements/year instead of ≥3 measurements/year).

### Statistical methods

Baseline was defined as the date of first eGFR measurement after 1 January 2004. Logistic regression models were used to assess the association between the different RP definitions and well established traditional renal risk factors - age, hypertension and diabetes [23-29]. The aim was to ensure that the definition of RP eventually chosen was a suitable compromise between maximising the number of persons eligible to be assessed for RP and the strongest risk factor associations. The C-Statistic was used to determine how well the logistic regression models discriminate between those who experience RP and those who do not. The C-statistic takes values between 0.5 to 1, where 0.5 corresponds to the worst discriminant model (one that performs no better than chance), and 1 corresponds to a model that perfectly discriminates between individuals with and without the outcome [30].

### Sensitivity analyses

Gender and ethnicity were only considered in additional multivariable logistic regression models, both due to limited numbers of women and individuals of non-Caucasian origin, and also due to conflicting reports about the extent to which these factors are independent predictors of rapid decline in eGFR [10,11,19,28,31-33]. We also considered a composite endpoint of RP and all-cause mortality. Finally we investigated the number of individuals who would be considered to have RP if a 3 ml/min/1.73 m^2^ decline was considered instead of 5 ml/min/1.73 m^2^.

### Additional analyses

To check the robustness of our main and alternative RP definitions, we investigated the number of individuals considered to have experienced RP under each definition that went on to develop CKD. Follow-up started at the end of the time period used to define whether an individual experienced RP or not, and any events after this time were considered.

## Results

Of 24,799 individuals with normal baseline eGFR, 22,603 (9.1%) had at least three eGFR measurements after 1 January 2004 and could potentially be assessed for RP. A total of 22,603 individuals had normal baseline eGFR ≥90 ml/min/1.73 m^2^, with at least three eGFR measurements after 1 January 2004 and could potentially be assessed for RP. The median number of measurements and length of follow-up was 12 (interquartile range (IQR) 7–16)) and 4.6 (IQR 2.7-6.1) years respectively. The median number of eGFR measurements available per individual per year was 3 (IQR 2–3) years.

### Baseline characteristics

Table 1 shows the baseline characteristics of individuals included in the analysis. Most of the 22,603 individuals eligible for inclusion in the analysis were male (72.7%), of Caucasian (46.8%) or unknown ethnicity (43.0%), less than 50 years old (90.1%), with BMI in the range of ≥18 to <26 kg/m^2^ (70.2%), current smokers (42.2%) or ex-smokers (17.4%) and with a current CD4 count ≥350 cell/mm^3^ (65.1%).

**Table 1:** **Baseline**^**a **^**characteristics of patients included in analyses of rapid progression, according to availability of data**

		**Patients with eGFR data available**	**≥4 years follow-up**			**≥3 years follow-up**		
			**Unrestricted**^**f**^	**≥3 eGFR per year**	**≥2 eGFR per year**	Unrestricted*	**≥3 eGFR per year**	**≥2 eGFR per year**
Eligible patients	Number (%)	22603 (100)	13061 (57.8)	3655 (16.2)	8298 (36.7)	16165 (71.5)	6375 (28.2)	12283 (54.3)
Average eGFR	Median (IQR)	109 (99–123)	108 (99–122)	107 (98–121)	108 (99–121)	109 (99–122)	108 (99–122)	108 (99–122)
eGFR >120 ml/min	Number (%)	6613 (29.3)	3616 (27.7)	943 (26)	225 (27)	4562 (28.1)	1723 (27)	3412 (28)
Gender	Male	16438 (72.7)	9335 (71.5)	2622 (71.7)	5999 (72.3)	11613 (71.8)	4617 (72.4)	8906 (72.5)
Race	White	10573 (46.8)	6343 (48.6)	1631 (44.6)	3835 (46.2)	7469 (46.2)	2584 (40.5)	5454 (44.4)
	Black	1806 (8.0)	1141 (8.7)	354 (9.7)	720 (8.7)	1346 (8.3)	581 (9.1)	1023 (8.3)
	Other	510 (2.3)	306 (2.3)	103 (2.8)	197 (2.4)	385 (2.4)	155 (2.4)	294 (2.4)
	Unknown	9714 (43.0)	5271 (40.4)	1567 (42.9)	3546 (42.7)	6965 (43.1)	3055 (47.9)	5512 (44.9)
Age group	>50 years	2238 (9.9)	1280 (9.8)	410 (11.2)	868 (10.5)	1641 (10.2)	702 (11.0)	1309 (10.7)
Hypertension^b^	Yes	2519 (11.1)	1526 (11.7)	494 (13.5)	1030 (12.4)	1865 (11.5)	784 (12.3)	1458 (11.9)
Diabetes^c^	Yes	659 (2.9)	428 (3.3)	145 (4.0)	290 (3.5)	517 (3.2)	237 (3.7)	413 (3.4)
Mode of HIV acquisition	Homosexual	10006 (44.3)	5642 (43.2)	1611 (44.1)	3710 (44.7)	7028 (43.5)	2876 (45.1)	5487 (44.7)
	IDU	3058 (13.5)	1783 (13.7)	450 (12.3)	1019 (12.3)	2196 (13.6)	740 (11.6)	1540 (12.5)
	Heterosexual	8095 (35.8)	4865 (37.3)	1385 (37.9)	3081 (37.1)	5952 (36.8)	2359 (37.0)	4499 (36.6)
	Other	1444 (6.4)	771 (5.9)	209 (5.7)	488 (5.9)	989 (6.1)	400 (6.3)	757 (6.2)
BMI at study entry (kg/m^2^)	<18	633 (2.8)	345 (2.6)	91 (2.5)	216 (2.6)	440 (2.7)	169 (2.7)	330 (2.7)
	≥18, <26	15865 (70.2)	9310 (71.3)	2542 (69.6)	5877 (70.8)	11473 (71.0)	4466 (70.1)	8717 (71.0)
	≥26, <30	3716 (16.4)	2231 (17.1)	677 (18.5)	1466 (17.7)	2727 (16.9)	1106 (17.4)	2088 (17.0)
	≥30	1423 (6.3)	845 (6.5)	238 (6.5)	536 (6.5)	1037 (6.4)	398 (6.2)	774 (6.3)
	Unknown	966 (4.3)	330 (2.5)	107 (2.9)	203 (2.5)	488 (3.0)	236 (3.7)	374 (3.0)
Hepatitis C^d^ positive	Yes	2765 (12.2)	1605 (12.3)	400 (10.9)	910 (11.0)	1958 (12.1)	653 (10.2)	1346 (10.6)
Hepatitis B^e^ positive	Yes	2773 (12.3)	1551 (12.4)	450 (12.3)	1001 (12.1)	1971 (12.2)	816 (12.8)	1501 (12.2)
Smoking status	Current	9544 (42.2)	5554 (42.5)	1467 (40.1)	3445 (41.5)	6930 (42.9)	2571 (40.3)	5198 (42.3)
	Previous	3930 (17.4)	2426 (18.6)	681 (18.6)	1557 (18.8)	2923 (18.1)	1193 (18.7)	2248 (18.3)
	Never	5824 (25.8)	3599 (27.6)	1086 (29.7)	2332 (28.1)	4314 (26.7)	1726 (27.1)	3299 (26.9)
	Unknown	3305 (14.6)	1482 (11.4)	421 (11.5)	964 (11.6)	1998 (12.4)	885 (13.9)	1538 (12.5)
Current CD4 count (cells/mm^3^)	<200	2823 (12.5)	1435 (11.0)	482 (13.2)	940 (11.3)	1831 (11.3)	833 (13.1)	1424 (11.6)
	200-349	4912 (21.7)	2797 (21.4)	826 (22.6)	1807 (21.8)	3427 (21.2)	1444 (22.7)	2641 (21.5)
	350-499	5565 (24.6)	3294 (25.2)	963 (26.4)	2109 (25.4)	4049 (25.1)	1632 (25.6)	3078 (25.1)
	≥500	9145 (40.5)	5492 (42.1)	1371 (37.5)	3417 (41.2)	6779 (41.9)	2432 (38.2)	5079 (41.4)
	Missing	158 (0.7)	43 (0.3)	13 (0.4)	25 (0.3)	79 (0.5)	34 (0.5)	61 (0.5)

*Abbreviations*: eGFR, estimated glomerular filtration rate; IDU, injecting drug user;

^a^Baseline was defined as the defined as the time, during prospective follow-up, of the first eGFR measurement on or after 1 January 2004.

^b^Hypertension was defined as a blood pressure of ≥150/≥100 mmHg or use of antihypertensive drugs and ace inhibitors.

^c^Diabetes was defined as receipt of antidiabetic treatment or verification of diabetes in a case report form.

^d^Hepatitis C was defined as detection of antibody to HCV plus detection or unknown presence of HCV RNA.

^e^Hepatitis B was defined as detection of HBV surface antigen, detection of HBV e antigen, or detection of HBV DNA plus antibody to HBV e antigen.

^f^Unrestricted was defined as no restriction concerning the number of eGFR measurements obtained per year.

There were 13,061 individuals with at least four years follow-up, and of these 3,655 (16.2% of the original 22,603 participants) had ≥3 measurements/year and 8,298 (36.7%) had ≥2 measurements/year. There were 16,165 individuals with at least three years of follow-up; of these 6,375 (28.2%) had ≥3 measurements/year, and 12,283 (54.3%) had ≥2 measurements/year (Table 1). Finally, 19,309 individuals had at least two years of follow-up; 10,756 (47.6%) had ≥3 measurements/year, and 17,211 (76.1%) had ≥2 measurements/year (Additional file 1: Table S1). The characteristics of those eligible for assessment for RP using the primary definition were similar to all included (Table 1), as for the other RP definitions based on varying follow-up periods and number of eGFR measurements/year.

### Percentage experiencing RP

**Figure 3:**
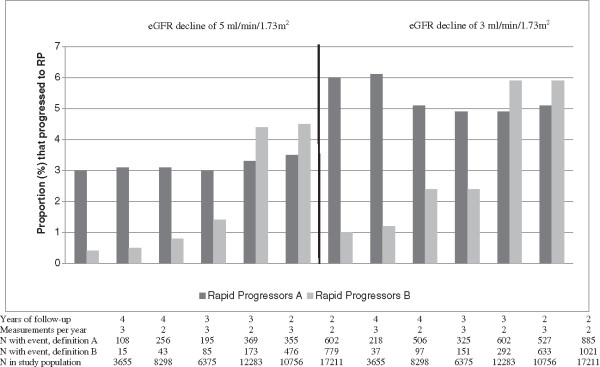
**Proportion of patients that satisfy different RP definitions, for decline of 5 or 3 ml/min/1.73 m**^**2**^**.**

The average rate of eGFR decline for all participants was -3.91 ml/min/1.73 m^2^ /year (95% confidence interval; -11.7, 3.8 ml/min/1.73 m^2^/year). Of the 3,655 individuals with ≥ 4 four years’ follow-up and ≥3 measurements/year, 108 (3.0%; Figure 3) experienced RP according to definition A. Similar proportions were considered to have experienced RP when considering shorter follow-up periods of three years (195/6375; 3.1%) and two years (355/10756; 3.3%). In contrast under RP definition B, considerably greater proportions were considered to have experienced RP regardless of whether two (476/10756; 4.4%), three (48/6375; 0.8%) or four (15/3655; 0.4%) years’ follow-up was considered.

When two annual eGFR measurements were required instead of three, similar proportions experienced RP under definition A, irrespective of the length of follow-up period (256/8298 (3.1%) experienced RP using four years follow-up, 369/12283 (3.0%) using three years follow-up, and 602/17211 (3.5%) using two years follow-up). The same was also true for RP definition B. Thus, it appears that the requirement for two or three eGFR measurements per year did not impact greatly on the proportion of individuals considered to be rapid progressors. When using a decline of 3 ml/min/1.73 m^2^ instead of 5 ml/min/1.73 m^2^ to define RP, the percentage of individuals that progressed to RP increased as expected across all strata (Figure 3).

### Association between traditional renal risk factors and rapid progression (RP)

In a multivariable logistic regression, the odds ratios (OR) for the association between age >50 years and RP were strong for every definition (Table 2); they varied between 1.90 to 2.54 for the primary definition (RP definition A) and between 2.55 to 2.80 for RP definition B. In contrast, there was more variation for the association with hypertension, and the strength depended on the length of follow-up. No associations between hypertension and RP were found when two years of follow-up was considered (the ORs varied between 0.99 and 1.14), whereas for three and four years of follow-up an association was generally found, although not all reached statistical significance at the 5% level. No RP definition demonstrated a clear association with diabetes (ORs varied between 0.48 to 1.24).

**Table 2:** **Associations**^**$ **^**between different rapid progression of renal function definitions and traditional renal disease risk factors**

		**With at least three eGFR measurements**				**With at least two eGFR measurements**			
**Years of follow-up**	**Baseline variables**	**RP definition A**		**RP definition B**		**RP definition A**		**RP definition B**	
		**OR (95% CI)**	**P**	**OR (95% CI)**	**P**	**OR (95% CI)**	**P**	**OR (95% CI)**	**P**
Four	Age >50 years	1.90 (1.15-3.14)	0.01	*		2.33 (1.69-3.20)	<0.001	2.65 (1.27-5.53)	0.01
	Hypertension	1.42 (0.85-2.37)	0.19			1.41 (1.00-1.97)	0.05	1.31 (0.55-3.10)	0.54
	Diabetes	1.06 (0.44-2.54)	0.90			0.88 (0.47-1.60)	0.68	0.48 (0.06-3.62)	0.48
Three	Age >50 years	1.91 (1.31-2.77)	<0.001	2.75 (1.64-4.59) <0.001		2.07 (1.58-2.71)	<0.001	2.80 (1.95-4.04)	<0.001
	Hypertension	1.58 (1.08-2.33)	0.02	1.31 (0.71-2.40) 0.39		1.39 (1.04-1.85)	0.03	0.79 (0.49-1.28)	0.34
	Diabetes	0.91 (0.45-1.83)	0.64	1.16 (0.45-3.00) 0.76		0.94 (0.56-1.59)	0.82	1.24 (0.62-2.51)	0.54
Two	Age >50 years	2.32 (1.78-3.04)	<0.001	2.70 (2.15-3.39) <0.001		2.54 (2.06-3.12)	<0.001	2.55 (2.07-3.14)	<0.001
	Hypertension	1.09 (0.80-1.50)	0.58	1.14 (0.87-1.49) 0.36		0.99 (0.78-1.27)	0.96	1.00 (0.78-1.28)	0.99
	Diabetes	0.92 (0.53-1.61)	0.78	0.73 (0.43-1.23) 0.24		1.14 (0.67-1.95)	0.62	0.98 (0.64-1.51)	0.93

RP definition A: First eGFR >90; average slope during follow-up ≥5 per year; absolute decline ≥5 in two consecutive years; last eGFR <90.

RP definition B: First eGFR >90; absolute decline ≥5 in all years of follow-up; last eGFR <90.

^$^Results from multivariable logistic regression analysis.

*Insufficient numbers experiencing RP with this definition.

The C-Statistics for these logistic regression models were very similar in magnitude, ranging from 0.56 to 0.62, for both definitions A and B. Addition of gender and ethnicity to these models did not substantively change the results (results not shown). The composite endpoint of RP and mortality markedly increased the number of events as expected, but the association with age and hypertension in the logistic regression remained similar (results not shown).

### Progression to CKD

For the primary definition of RP (definition A with 4 years’ follow-up and at least 3 measurements per year), 13 (12%) individuals who experienced RP progressed to CKD by the end of the follow-up period (i.e. the test had a positive predictive value [PPV] of 12%), whereas only 21 (0.6%) of those without RP progressed to CKD (i.e. one minus the negative predictive value [NPV] was 0.6%, or NPV = 99.4%; Table 3). These values corresponded to sensitivity and specificity for the primary definition of 38.2% and 97.4%, suggesting RP from initial normal eGFR levels is not a good marker for subsequent CKD, within the timeframes analysed in this study. As the definition of RP became less restrictive (with fewer years of follow-up and number of eGFR measurements/year), fewer of those who experienced RP progressed to CKD (PPV estimates decreased from 12% to 3.3%), whereas the proportion of those not experiencing RP, who did progress to CKD remained constant (1-NPV varied from 0.4% to 0.6%). For RP definition B, the PPV and sensitivity estimates were generally smaller compared to obtained from definition A, and the specificity and (1-NPV) estimates were similar. When using an eGFR decline of 3 instead of 5 ml/min/1.73 m^2^ to define RP, the proportion that progressed to CKD (i.e. the PPV) decreased (Table 3). Much weaker associations were seen when an endpoint of CKD or death was considered (Additional file 2: Table S2).

**Table 3:** Percentage who progressed to incident CKD*, according to different definitions of RP

**Years of follow-up considered**	**Number of eGFR per year**	**Excluded in analysis**	**RP definition A**				**RP definition B**			
			^**a**^**RP (PPV)**	^**b**^**non-RP (1-NPV)**	**Sensitivity (%)**	**Specificity (%)**	**RP (PPV)**	**non-RP (1-NPV)**	**Sensitivity (%)**	**Specificity (%)**
			**Declines of >5 ml/min/1.73 m**^**2**^**required to meet the RP definition**							
Four	Three	65/18948 (0.3)	13/108(12.0)	21/3547 (0.6)	38.2	97.4	0/15 (0.0)	34/3640 (0.9)	0.0	99.6
Four	Two	42/14305 (0.3)	23/256 (9.0)	34/8042 (0.4)	40.4	97.2	2/43 (4.7)	55/8255 (0.7)	3.5	99.5
Three	Three	47/16228 (0.3)	15/195 (7.7)	37/6180 (0.6)	28.8	97.2	4/85 (4.7)	48/6290 (0.8)	7.7	98.7
Three	Two	24/10320 (0.2)	20/369 (5.4)	55/11914 (0.5)	26.7	97.1	7/173 (4.1)	68/12110 (0.6)	9.3	98.6
Two	Three	28/11847 (0.2)	14/355 (3.9)	57/10401 (0.6)	19.7	96.8	16/476 (3.4)	55/10280 (0.5)	22.5	95.7
Two	Two	7/5392 (0.1)	20/602 (3.3)	72/16609 (0.4)	21.7	96.6	23/779 (3.0)	69/16432 (0.4)	25.0	95.6
			**Declines of >3 ml/min/1.73 m**^**2**^** required to meet the RP definition**							
Four	Three	65/18948 (0.3)	18/218 (8.3)	16/3437 (0.5)	52.9	94.5	3/37 (8.1)	31/3618 (0.9)	8.8	99.1
Four	Two	42/14305 (0.3)	27/506 (5.3)	30/7792 (0.4)	47.4	94.2	6/97 (6.2)	51/8201 (0.6)	10.5	98.9
Three	Three	47/16228 (0.3)	21/325 (6.5)	31/6050 (0.5)	40.4	95.2	8/151 (0.7)	44/6224 (27.5)	15.4	97.7
Three	Two	24/10320 (0.2)	25/602 (4.2)	50/11681 (0.4)	33.3	95.3	14/292 (4.8)	61/11991 (0.5)	18.7	97.7
Two	Three	28/11847 (0.2)	20/527 (3.8)	51/10229 (0.5)	28.2	95.3	22/633 (2.8)	49/10123 (0.5)	31.0	94.3
Two	Two	7/5392 (0.1)	30/885 (3.4)	62/16326 (0.4)	32.6	95.0	32/1021(4.5)	60/16190 (0.4)	34.8	94.2

*Two consecutive eGFR measurements <60 ml/min/1.73 m^2^.

^a^RP: Rapid Progressors who developed CKD (i.e., the positive predictive value (PPV)).

^b^Non-RP: non-Rapid Progressors who developed CKD (i.e., 1-negative predictive value (NPV)).

## Discussion

This manuscript considered an operational definition of RP in HIV-positive individuals. Our results suggest a definition that would apply to individuals with normal baseline eGFR (≥90 ml/min/1.73 m^2^) with at least three years’ follow-up and at least two eGFR measurements per year, for the reasons described in subsequent paragraphs. Rapid progressors are defined as those who had: (i) an average decline over the three-year period of ≥5 ml/min/m^2^ per year, (ii) an eGFR decline ≥5 ml/min/1.73 m^2^ in two consecutive years and (iii) an eGFR at the end of the three year period of <90 ml/min/1.73 m^2^ (RP definition A). This definition allows inclusion of a reasonable number of individuals that are broadly representative of the study cohort. It also had reasonable discriminatory ability to identify those who would go on to develop incident CKD, although none demonstrated particularly strong prognostic ability. However, as follow up after having experienced RP is still relatively short, later studies will show if those experiencing RP will be at higher risk of CKD and mortality in the longer term [34].

In deriving a definition for RP, we considered an annual decline ≥5 ml/min/1.73 m^2^ instead of the ≥3 ml/min/1.73 m^2^ decline as previously reported [13-18]. This ensured that only clinically relevant events were identified by reducing the impact of the “noise” that is introduced by random variability in eGFR measurements [9] and secondly this cut-off agrees with new guidelines from KDIGO [12]. When we considered a cut-off of ≥3 ml/min/1.73 m^2^ in sensitivity analyses, we found a much larger proportion defined as having RP, weaker associations with traditional renal risk factors, and less predictive ability with respect to future development of CKD. Additionally, the KDIGO guidelines do not specify progression in those without pre-existing CKD, which is the study population included in this present analysis. Limiting the definition to those whose final eGFR is outside of the normal range (i.e. <90 ml/min/1.73 m^2^) should hopefully ensure that any changes in eGFR are clinically relevant.

RP definition A gave more consistent percentages than RP definition B, where the percentage considered as RPs varied from 0.4% to 4.5%, as shown in Figure 3, depending on the length of follow-up and number of measurements per year considered. Furthermore, a higher percentage of those with RP under definition A progressed to CKD compared to definition B. As RP definition A combines absolute and average renal function decline, it is based on all available eGFR and is less restrictive (in particular for longer follow-up) and therefore may better reflect the dynamics of RP than definition B. One potential limitation of our RP definition is that we did not base our definitions on relative (percentage) changes. There have been suggestions that a 25% decline from baseline in eGFR levels may be an appropriate definition for RP [12].

For individuals with at least two eGFR measurements per year, requirement of at least three years of follow-up captures more eligible individuals into the RP analyses compared to those with four years of follow-up, which was the follow-up period considered in our original primary definition. Shorter periods of follow-up with more included individuals are more likely to ensure a more inclusive study population and more statistical power to identify associations. Clearly, the longer the follow-up period, the more likely we are to introduce selection bias, with those with the worst predicted outcome being excluded if they had died or dropped out of the study due to ill health. Therefore, the choice of two or three years’ follow-up could be taken in an attempt to minimise selection bias, although one cannot ever definitively exclude this. Conversely, two years may be too short a time period to permit accurate determination of RP,therefore, three years appeared most suitable.

Requirement of three rather than two eGFR measurements/year provides a more reliable slope and identifies a greater proportion progressing to CKD. Two measurements per year may however, be more applicable in routine clinical care, particularly as there is a move towards less frequent laboratory monitoring of HIV positive individuals. In this study there was a median of three creatinine measurements per individual per year, which means that any definition of RP that requires three or more measurements performed each year is unlikely to be operational due to high proportions being excluded.

In our definition of RP, the proportion of RPs who progressed to CKD were ten–fold higher than non-RPs who progressed to CKD, though the sensitivity for these results were low, regardless of the definition used. This suggests that RP is not necessarily predictive of incident CKD, perhaps because treatment switches away from potentially nephrotoxic drugs are made before incident CKD has occurred [32] or because longer follow-up would be needed to detect subsequent CKD events.

We used a small and selective number of variables, defined a priori, to investigate their association with each of our definitions of RP [23-29]. Our current analysis found the strong association between age and hypertension with RP when using our primary definition A. In contrast, it was difficult to reach any conclusions for diabetes, where associations were small or suggested a protective effect. Addition of gender and ethnicity to the logistic model made no changes to the results [10,17,19,21,28,31-33].

A composite endpoint of RP and all-cause mortality provided more endpoints as expected, but a similar magnitude of the association between age, hypertension and diabetes. As very few deaths in HIV-positive individuals were attributable to renal disease, including all-cause mortality in the endpoint did not give a more sensible definition of RP. Therefore, we would not recommend using a combined endpoint of all-cause mortality and RP.

In this analysis we have not considered exposure to antiretroviral treatment, as our aim was not to study factors associated with RP, but rather to develop a definition of RP that can be used in the future in such studies. Scherzer et al. demonstrated that tenofovir use was associated with rapid decline in renal function, where rapid decline was defined as an annual decline of 3 ml/min/1.73 m^2^ for two consecutive years, but not specified if absolute or average [18].

Sensitivity analyses with eGFR calculated from the MDRD or CKD-EPI equations [35,36], were not carried out, as both of these methods required ethnicity, which some of the participating cohorts in the D:A:D study are prohibited from collecting. However, recent studies have suggestedthat consistent results are obtained regardless of the estimation used [37,38], although other studies have not found this [38-40]. Furthermore, the CG formula was adjusted for BSA, in line with previous D:A:D analyses [2] and there are conflicting reports regarding whether this is appropriate [41,42]. Analyses performed on this cohort on standardising for BSA suggests that it has little impact on obtained eGFRs (Prof A Mocroft, personal communication).

In the D:A:D study, no systematic information is collected on proteinuria, which is a reliable marker for rapid eGFR decline [31]. Furthermore, the C-Statistic values obtained were in the range of 0.56 to 0.62 and so the ability of the logistic model to discriminate between those with and without RP would only be considered ‘satisfactory’ using Hosmer and Lemeshow’s criteria [43]. Using multiple endpoints with different length of follow-up and number of measurements in order to identify a model that best fits our selected variables associated with RP may lead to us finding a false positive result.

## Conclusions

In this analysis, we have developed a standardised operational definition for rapid progression of eGFR. We believe this definition balances practical issues regarding the data likely to be available in standard HIV observational databases with a definition that has clinical relevance. Under this definition, 3% of those with normal eGFR went on to develop RP (definition A), and of these 5.4% (20/369) progressed to CKD during a median of 5.6 years of follow-up, including the follow-up used to define individuals as experiencing RP or not.

Despite the weak association between our RP definition and subsequent development of CKD, perhaps due to the limited follow-up available, other studies have shown that short-term changes in eGFR relate to subsequent mortality and underline the clinical importance of RP. In future analyses, the D:A:D study group will consider this definition of RP to identify and assess potential risk factors including antiretroviral use, as a dynamic tool to be used in addition to commonly used CKD analysis.

## Competing interests

D Kamara, L. Ryom, J.D. Lundgren and M. Ross have no conflicts of interest.

O. Kirk had prior/present board membership at ViiV Healthcare, Gilead Sciences and Merck, received payment for lectures and/or for development of educational presentations from Abbott, Gilead Sciences and Tibotec. Travel/accommodations/meeting expenses from Abbott, BMS, Gilead Sciences, Merck and ViiV Healthcare.

P. Reiss has served as a scientific advisor to Bristol-Myers Squibb, Gilead Sciences, Grupo Ferrer, GlaxoSmithKline, Janssen Pharmaceuticals, Merck & Co, Inc, and ViiV Healthcare. He has served on data and safety monitoring boards and endpoint adjudication committees for Janssen Pharmaceuticals and his institution has received honoraria for speaking engagements at scientific conferences from Bristol-Myers Squibb, Gilead Sciences, Inc, GlaxoSmithKline. He has received research support from Gilead Sciences, ViiV Healthcare, Merck & Co, Inc, Janssen Pharmaceuticals, Bristol-Myers Squibb, Abbott, and Boehringer Ingelheim Pharmaceuticals.

P. Morlat is board member at ViiV Healthcare, MSD, Gilead Sciences and Boehringer Ingelheim Pharmaceuticals and had expenses paid for travel/accommodation/meetings by BMS, ViiV Healthcare, Abbott and MSD.

O. Moranne has received honoraria speaker from Abbott and Gilead Sciences, is a board member for Roche and had expenses paid for travel/accommodation/meetings by Roche and Baxter companies.

C.A. Fux is an advisory board member for Gilead Sciences and MSD, has pending grants from Gilead Sciences and Abbott and received payment for lectures by Gilead HIV and the body.

A. Mocroft has received consultancy fees/honoraria/speaker fees from BMS, Pfizer, Merck, BI, and Gilead Sciences.

C. Sabin has received funding for Advisory Board membership, speaker panels and provision of educational materials for Gilead Sciences, Abbott Pharmaceuticals, Viiv, Merck Sharp & Dohme, Janssen-Cilag and Bristol-Myers Squibb.

C. Smith has a pending grant from Bristol-Myers Squibb and received payment for development of educational presentations by Gilead Sciences and ViiV Healthcare.

## Authors’ contributions

DK, LR, AM, OK, CS and JDL developed the initial study protocol. LR performed study co-ordination and prepared the datasets for analysis. DK performed the statistical analysis and DK and LR prepared the initial draft of the manuscript. All authors have provided input to the analyses, have contributed with data, have participated in the development of the manuscript and have seen and approved the final version.

## Authors’ information

David A Kamara and Lene Ryom Joint co-authors.

## Pre-publication history

The pre-publication history for this paper can be accessed here:

http://www.biomedcentral.com/1471-2369/15/51/prepub

## Supplementary Material

Additional file 1: Table S1 ^a^Baseline characteristics of patients included in analyses of rapid progression, according to availability of data. Click here for file

Additional file 2: Table S2 Percentage who progressed to incident CKD or death according to different definitions of RP. Click here for file

## D:a:D steering committee

Names marked with *, Chair with #

**Members of the D:A:D SC from the Oversight Committee:** B. Powderly*, N. Shortman*, C Moecklinghoff *, G Reilly*, X. Franquet* **D:A:D Central Coordination:** L. Ryom, C.A. Sabin*, D. Kamara, C. Smith, A. Phillips*, A. Mocroft, J. Tverland, M. Mansfeld, J. Nielsen, D. Raben, J.D. Lundgren#;

**D:A:D data managers:** R. Salbøl Brandt (coordinator), M. Rickenbach, I. Fanti, E. Krum, M. Hillebregt, S Geffard, A. Sundström, M. Delforge, E. Fontas, F. Torres, H. McManus, S. Wright, J. Kjær.

**Verification of Endpoints**: A. Sjøl (CVD primary endpoint), P. Meidahl (oncology, new endpoint), J. Helweg-Larsen (hematology, new endpoint), J. Schmidt Iversen (nephrology, new endpoint)

**Kidney working group:** L. Ryom, A. Mocroft, O. Kirk*, P. Reiss*, M. Ross, C.A. Fux, P. Morlat, O. Moranne, A.M. Kesselring, D.A. Kamara, C. Smith, J.D. Lundgren#

**Mortality working group:** C. Smith, L. Ryom, A. Phillips*, R. Weber* , P. Morlat, C. Pradier*, P. Reiss*, N. Friis-Møller, J. Kowalska, J.D. Lundgren#

**Cancer working group:** C. Sabin*, M. Law*, A . d’Arminio Monforte*, F. Dabis*, M. Bruyand, P. Reiss*, C. Smith, D.A. Kamara, M Bower, G. Fätkenheuer, A. Donald, A.Grulich, L.Ryom, J.D. Lundgren#

The members of the 11 Cohorts are as follows:

**ATHENA** (AIDS Therapy Evaluation Project Netherlands):

**Central coordination:** P. Reiss*, S. Zaheri, M. Hillebregt, L. Gras;

**Participating physicians** (¤Site coordinating physicians): **Academisch Medisch Centrum bij de Universiteit van Amsterdam, Amsterdam**: Prof. dr. J.M. Prins¤, Prof. dr. T.W. Kuijpers, Dr. H.J. Scherpbier, Dr. J.T.M. van der Meer, Dr. F.W.M.N. Wit, Dr. M.H. Godfried, Prof. dr. P. Reiss*, Prof. dr. T. van der Poll, Dr. F.J.B. Nellen, Prof. dr. J.M.A. Lange, Dr. S.E. Geerlings, Dr. M. van Vugt, Dr. D. Pajkrt, Drs. J.C. Bos, Drs. M. van der Valk, Drs. M.L. Grijsen, Dr. W.J. Wiersinga, Dr. A. Goorhuis, Dr. J.W.R. Hovius. **Academisch Ziekenhuis Maastricht, Maastricht**: Dr. S. Lowe¤, Dr. A. Oude Lashof, Dr. D. Posthouwer. **Catharina-ziekenhuis, Eindhoven**: Drs. M.J.H. Pronk¤, Dr. H.S.M. **Ammerlaan. Erasmus Medisch Centrum, Rotterdam**: Dr. M.E. van der Ende¤, Dr. T.E.M.S. de Vries-Sluijs, Dr. C.A.M. Schurink, Dr. J.L. Nouwen, Dr. A. Verbon, Drs. B.J.A. Rijnders, Dr. E.C.M. van Gorp, Drs. M. van der Feltz. **Erasmus Medisch Centrum–Sophia, Rotterdam**: Dr. G.J.A. Driessen, Dr. A.M.C. van Rossum. **Flevoziekenhuis. Almere**: Dr. J. Branger¤. **HagaZiekenhuis, Den Haag**: Dr. E.F. Schippers¤, Dr. C. van Nieuwkoop, Drs. E.P. van Elzakker. **Isala Klinieken, Zwolle**: Dr. P.H.P. Groeneveld¤, Drs. J.W. Bouwhuis. **Kennemer Gasthuis**: Drs. R. Soetekouw¤, Prof. dr. R.W. ten Kate. **Leids Universitair Medisch Centrum, Leiden**: Dr. F.P. Kroon¤, Prof. dr. J.T. van Dissel, Dr. S.M. Arend, Dr. M.G.J. de Boer, Drs. H. Jolink, Dr. H.J.M. ter Vollaard, Drs. M.P. Bauer. **Maasstadziekenhuis, Rotterdam**: Dr. J.G. den Hollander¤, Dr. K. Pogany. **Medisch Centrum Alkmaar, Alkmaar**: Drs. G. van Twillert¤, Drs. W. Kortmann¤, Dr. J.W.T. Cohen Stuart, Dr. B.M.W. Diederen. **Medisch Centrum Haaglanden, Den Haag**: Dr. E.M.S. Leyten¤, Dr. L.B.S. Gelinck. **Medisch Spectrum Twente, Enschede**: Drs. G.J. Kootstra¤, Drs. C.E. Delsing. **Onze Lieve Vrouwe Gasthuis, Amsterdam**: Prof. dr. K. Brinkman¤, Dr. W.L. Blok, Dr. P.H.J. Frissen, Drs. W.E.M. Schouten, Drs. G.E.L. van den Berk. **Sint Elisabeth Ziekenhuis, Tilburg**: Dr. M.E.E. van Kasteren¤, Dr. A.E. Brouwer. **Sint Lucas Andreas Ziekenhuis, Amsterdam**: Dr. J. Veenstra¤, Dr. K.D. Lettinga. **Slotervaartziekenhuis, Amsterdam**: Dr. J.W. Mulder¤, Drs. S.M.E. Vrouenraets, Dr. F.N. Lauw. **Stichting Medisch Centrum Jan van Goyen, Amsterdam**: Drs. A. van Eeden¤, Dr. D.W.M. Verhagen. **Universitair Medisch Centrum Groningen, Groningen**: Drs. H.G. Sprenger¤, Drs. R. Doedens, Dr. E.H. Scholvinck, Dr. S. van Assen, Dr. W.F.W. Bierman. **Universitair Medisch Centrum Sint Radboud, Nijmegen**: Dr. P.P. Koopmans¤, Dr. M. Keuter, Dr. A.J.A.M. van der Ven, Dr. H.J.M. ter Hofstede, Dr. A.S.M. Dofferhoff, Dr. A Warris, Dr. R. van Crevel. **Universitair Medisch Centrum Utrecht, Utrecht**: Prof. dr. A.I.M. Hoepelman¤, Dr. T. Mudrikova, Dr. M.M.E. Schneider, Dr. P.M. Ellerbroek, Dr. J.J. Oosterheert, Dr. J.E. Arends, Dr. M.W.M. Wassenberg, Dr. R.E. Barth. **Vrije Universiteit Amsterdam, Amsterdam**: Dr. M.A. van Agtmael¤, Dr. R.M. Perenboom, Drs. F.A.P. Claessen, Dr. M. Bomers, Dr. E.J.G. Peters. **Wilhelmina Kinderziekenhuis, Utrecht**: Dr. S.P.M. Geelen, Dr. T.F.W. Wolfs, Dr. L.J. Bont. **Ziekenhuis Rijnstate, Arnhem**: Dr. C. Richter¤, Dr. J.P. van der Berg, Dr. E.H. Gisolf. **Admiraal De Ruyter Ziekenhuis, Vlissingen**: Drs. M. van den Berge¤, Drs. A. Stegeman. **Medisch Centrum Leeuwarden, Leeuwarden**: Dr. M.G.A. van Vonderen¤, Drs. D.P.F. van Houte. **Medisch Centrum Zuiderzee, Lelystad**: Dr. S. Weijer¤, Dr. R. el Moussaoui. **Sint Elisabeth Hospitaal, Willemstad - Curaçao**: Dr. C. Winkel, Drs. F. Muskiet, Drs. Durand, Drs. R. Voigt.

**Aquitaine Cohort** (France):

**Composition of the GECSA: Coordination:** F. Dabis***Epidemiology and Methodology:** M. Bruyand, G. Chêne, F. Dabis, S. Lawson-Ayayi, R. Thiébaut. **Infectious Diseases and Internal Medicine:** F. Bonnal, F. Bonnet, N. Bernard, L. Caunègre, C. Cazanave, J. Ceccaldi, D. Chambon, I. Chossat, FA. Dauchy, S. De Witte, M. Dupon, P. Duffau, H. Dutronc, S. Farbos, V. Gaborieau, MC. Gemain, Y. Gerard, C. Greib, M. Hessamfar, D. Lacoste, P. Lataste, S. Lafarie, E. Lazaro, D. Malvy, JP. Meraud, P. Mercié, E. Monlun, P. Morlat, D. Neau, A. Ochoa, JL. Pellegrin, T. Pistone, JM. Ragnaud, MC. Receveur, S. Tchamgoué, MA. Vandenhende, JF. Viallard. **Immunology:** JF. Moreau, I. Pellegrin. **Virology:** H. Fleury, ME. Lafon, B. Masquelier, P. Trimoulet. **Pharmacology**: D. Breilh. **Drug monitoring:** F. Haramburu, G. Miremont-Salamé. **Data collection and processing:** MJ. Blaizeau, M. Decoin, J. Delaune, S. Delveaux, C. D’Ivernois, C. Hanapier, O. Leleux, B. Uwamaliya-Nziyumvira, X Sicard. **Computing and Statistical analysis:** S. Geffard, G. Palmer, D. Touchard. **Scientific committee:** F. Bonnet, M. Dupon, P. Mercié, P. Morlat, JL. Pellegrin, JM. Ragnaud, F. Dabis*.

**AHOD** (Australian HIV Observational Database, Australia):

**Central coordination**: M. Law* , K. Petoumenos, H. McManus, S. Wright, C. Bendall (Sydney, New South Wales);

**Participating physicians** (city, state): R. Moore, S. Edwards, J. Hoy, K. Watson, N. Roth, J. Nicholson (Melbourne, Victoria); M Bloch, T. Franic, D. Baker, R. Vale, A. Carr, D. Cooper (Sydney, New South Wales); J. Chuah, M. Ngieng (Gold Coast, Queensland), D. Nolan, J. Skett (Perth, Western Australia).

**BASS** (Spain):

**Central coordination**: G. Calvo*, F. Torres, S. Mateu (Barcelona);

**Participating physicians** (city): P. Domingo, M.A. Sambeat, J. Gatell, E. Del Cacho, J. Cadafalch, M. Fuster (Barcelona); C. Codina, G. Sirera, A. Vaqué (Badalona).

**The Brussels St Pierre Cohort** (Belgium):**Coordination:** S. De Wit*, N. Clumeck, M. Delforge, C. Necsoi. **Participating physicians:** N. Clumeck, S. De Wit*, AF Gennotte, M. Gerard, K. Kabeya, D. Konopnicki, A. Libois, C. Martin, M.C. Payen, P. Semaille, Y. Van Laethem.

**CPCRA** (USA):

**Central coordination**: J. Neaton, G. Bartsch, W.M. El-Sadr*, E. Krum, G. Thompson, D. Wentworth; **Participating physicians** (city, state): R. Luskin-Hawk (Chicago, Illinois); E. Telzak (Bronx, New York); W.M. El-Sadr (Harlem, New York); D.I. Abrams (San Francisco, California); D. Cohn (Denver, Colorado); N. Markowitz (Detroit, Michigan); R. Arduino (Houston, Texas); D. Mushatt (New Orleans, Louisiana); G. Friedland (New Haven, Connecticut); G. Perez (Newark, New Jersey); E. Tedaldi (Philadelphia, Pennsylvania); E. Fisher (Richmond, Virginia); F. Gordin (Washington, DC); L.R. Crane (Detroit, Michigan); J. Sampson (Portland, Oregon); J. Baxter (Camden, New Jersey).

**EuroSIDA (multinational) Coordinating Centre:**: J Lundgren*#, O Kirk*, A Mocroft, A Cozzi-Lepri, D Grint, D Podlekareva, J Kjær, L Peters, J Reekie, J Kowalska, J Tverland, A H Fischer, J Nielsen **Participating countries and physicians**:

**Argentina**: (M Losso), C Elias, Hospital JM Ramos Mejia, Buenos Aires. **Austria**: (N Vetter), Pulmologisches Zentrum der Stadt Wien, Vienna; R Zangerle, Medical University Innsbruck, Innsbruck.

**Belarus**: (I Karpov), A Vassilenko, Belarus State Medical University, Minsk, VM Mitsura, Gomel State Medical University, Gomel; O Suetnov, Regional AIDS Centre, Svetlogorsk. **Belgium**: (N Clumeck), S De Wit*, M Delforge, Saint-Pierre Hospital, Brussels; R Colebunders, Institute of Tropical Medicine, Antwerp; L Vandekerckhove, University Ziekenhuis Gent, Gent. **Bosnia-Herzegovina**: (V Hadziosmanovic), Klinicki Centar Univerziteta Sarajevo, Sarajevo. **Bulgaria**: (K Kostov), Infectious Diseases Hospital, Sofia.

**Croatia**: (J Begovac), University Hospital of Infectious Diseases, Zagreb.

**Czech Republic**: (L Machala), D Jilich, Faculty Hospital Bulovka, Prague; D Sedlacek, Charles University Hospital, Plzen.

**Denmark**: (J Nielsen), G Kronborg,T Benfield, M Larsen, Hvidovre Hospital, Copenhagen; J Gerstoft, T Katzenstein, A-B E Hansen, P Skinhøj, Rigshospitalet, Copenhagen; C Pedersen, Odense University Hospital, Odense; L Ostergaard, Skejby Hospital, Aarhus.

**Estonia**: (K Zilmer), West-Tallinn Central Hospital, Tallinn; Jelena Smidt, Nakkusosakond Siseklinik, Kohtla-Järve.

**Finland**: (M Ristola), Helsinki University Central Hospital, Helsinki.

**France**: (C Katlama), Hôpital de la Pitié-Salpétière, Paris; J-P Viard, Hôpital Necker-Enfants Malades, Paris; P-M Girard, Hospital Saint-Antoine, Paris; JM Livrozet, Hôpital Edouard Herriot, Lyon; P Vanhems, University Claude Bernard, Lyon; C Pradier, Hôpital de l’Archet, Nice; F Dabis*, D Neau, Unité INSERM, Bordeaux.

**Germany**: (J Rockstroh), Universitäts Klinik Bonn; R Schmidt, Medizinische Hochschule Hannover; J van Lunzen, O Degen, University Medical Center Hamburg-Eppendorf, Infectious Diseases Unit, Hamburg; HJ Stellbrink, IPM Study Center, Hamburg; S Staszewski, JW Goethe University Hospital, Frankfurt; M Bickel, Medizinische Poliklinik, Munich; G. Fätkenheuer, Universität Köln, Cologne.

**Greece**: (J Kosmidis), P Gargalianos, G Xylomenos, J Perdios, Athens General Hospital; G Panos, A Filandras, E Karabatsaki, 1st IKA Hospital; H Sambatakou, Ippokration Genereal Hospital, Athens.

**Hungary**: (D Banhegyi), Szent Lásló Hospital, Budapest.

**Ireland**: (F Mulcahy), St. James’s Hospital, Dublin.

**Israel**: (I Yust), D Turner, M Burke, Ichilov Hospital, Tel Aviv; S Pollack, G Hassoun, Rambam Medical Center, Haifa; S Maayan, Hadassah University Hospital, Jerusalem.

**Italy**: (S Vella), Istituto Superiore di Sanità, Rome; R Esposito, I Mazeu, C Mussini, Università Modena, Modena; C Arici, Ospedale Riuniti, Bergamo; R Pristera, Ospedale Generale Regionale, Bolzano; F Mazzotta, A Gabbuti, Ospedale S Maria Annunziata, Firenze; V Vullo, M Lichtner, University di Roma la Sapienza, Rome; A Chirianni, E Montesarchio, M Gargiulo, Presidio Ospedaliero AD Cotugno, Monaldi Hospital, Napoli; G Antonucci, A Testa, P Narciso, C Vlassi, M Zaccarelli, Istituto Nazionale Malattie Infettive Lazzaro Spallanzani, Rome; A Lazzarin, A Castagna, N Gianotti, Ospedale San Raffaele, Milan; M Galli, A Ridolfo, Osp. L. Sacco, Milan; A d’Arminio Monforte, Istituto Di Clinica Malattie Infettive e Tropicale, Milan.

**Latvia**: (B Rozentale), I Zeltina, Infectology Centre of Latvia, Riga.

**Lithuania**: (S Chaplinskas), Lithuanian AIDS Centre, Vilnius.

**Luxembourg**: (R Hemmer), T Staub, Centre Hospitalier, Luxembourg.

**Netherlands**: (P Reiss*), Academisch Medisch Centrum bij de Universiteit van Amsterdam, Amsterdam. **Norway**: (V Ormaasen), A Maeland, J Bruun, Ullevål Hospital, Oslo.

**Poland**: (B Knysz) J Gasiorowski, Medical University, Wroclaw; A Horban, E Bakowska, Centrum Diagnostyki i Terapii AIDS, Warsaw; A Grzeszczuk, R Flisiak, Medical University, Bialystok; A Boron-Kaczmarska, M Pynka, M Parczewski, Medical Univesity, Szczecin; M Beniowski, E Mularska, Osrodek Diagnostyki i Terapii AIDS, Chorzow; H Trocha, Medical University, Gdansk; E Jablonowska, E Malolepsza, K Wojcik, Wojewodzki Szpital Specjalistyczny, Lodz.

**Portugal**: (F Antunes), M Doroana, L Caldeira, Hospital Santa Maria, Lisbon; K Mansinho, Hospital de Egas Moniz, Lisbon; F Maltez, Hospital Curry Cabral, Lisbon.

**Romania**: (D Duiculescu), Spitalul de Boli Infectioase si Tropicale: Dr. Victor Babes, Bucarest.

**Russia**: (A Rakhmanova), Medical Academy Botkin Hospital, St Petersburg; N Zakharova, St Petersburg AIDS Centre, St Peterburg; S Buzunova, Novgorod Centre for AIDS, Novgorod.

**Serbia**: (D Jevtovic), The Institute for Infectious and Tropical Diseases, Belgrade.

**Slovakia**: (M Mokráš), D Staneková, Dérer Hospital, Bratislava.

**Slovenia**: (J Tomazic), University Clinical Centre Ljubljana, Ljubljana.

**Spain**: (J González-Lahoz), V Soriano, P Labarga, J Medrano, Hospital Carlos III, Madrid; S Moreno, JM Rodriguez, Hospital Ramon y Cajal, Madrid; B Clotet, A Jou, R Paredes, C Tural, J Puig, I Bravo, Hospital Germans Trias i Pujol, Badalona; JM Gatell, JM Miró, Hospital Clinic i Provincial, Barcelona; P Domingo, M Gutierrez, G Mateo, MA Sambeat, Hospital Sant Pau, Barcelona.

**Sweden**: (A Karlsson), Venhaelsan-Sodersjukhuset, Stockholm; L Flamholc, Malmö University Hospital, Malmö.

**Switzerland**: (B Ledergerber), R Weber*, University Hospital, Zürich; P Francioli, M Cavassini, Centre Hospitalier Universitaire Vaudois, Lausanne; B Hirschel, E Boffi, Hospital Cantonal Universitaire de Geneve, Geneve; H Furrer, Inselspital Bern, Bern; M Battegay, L Elzi, University Hospital Basel.

**Ukraine**: (E Kravchenko), N Chentsova, Kiev Centre for AIDS, Kiev; V Frolov, G Kutsyna, Luhansk State Medical University; Luhansk; S Servitskiy, Odessa Region AIDS Center, Odessa; M Krasnov, Kharkov State Medical University, Kharkov.

**United Kingdom**: (S Barton), St. Stephen’s Clinic, Chelsea and Westminster Hospital, London; AM Johnson, D Mercey, Royal Free and University College London Medical School, London (University College Campus); A Phillips, MA Johnson, A Mocroft, Royal Free and University College Medical School, London (Royal Free Campus); M Murphy, Medical College of Saint Bartholomew’s Hospital, London; J Weber, G Scullard, Imperial College School of Medicine at St. Mary’s, London; M Fisher, Royal Sussex County Hospital, Brighton; C Leen, Western General Hospital, Edinburgh.

**HivBivus** (Sweden):

**Central coordination**: L. Morfeldt*, G. Thulin, A. Sundström.

**Participating physicians (city)**: B. Åkerlund (Huddinge); K. Koppel, A. Karlsson (Stockholm); L. Flamholc, C. Håkangård (Malmö).

**The ICONA Foundation** (Italy): **BOARD OF DIRECTORS** M. Moroni (Chair), G. Angarano, A. Antinori, , O. Armignacco, A. d’Arminio Monforte*, F. Castelli, R. Cauda, , G. Di Perri, M. Galli, R. Iardino, G. Ippolito, A. Lazzarin, C.F. Perno, F. von Schloesser, P. Viale **SCIENTIFIC SECRETARY** A d’Arminio Monforte*, A. Antinori, A. Castagna, F. Ceccherini-Silberstein, A. Cozzi-Lepri, E. Girardi, S. Lo Caputo, C. Mussini, M. Puoti **ICONA STEERING COMMITTEE** Massimo Andreoni, Adriana Ammassari, Andrea Antinori, Antonella d’Arminio Monforte, Claudia Balotta, Paolo Bonfanti, Stefano Bonora, Marco Borderi, MRosaria Capobianchi, Antonella Castagna, Francesca Ceccherini-Silberstein, Antonella Cingolani, Paola Cinque, Alessandro Cozzi-Lepri, Antonella d’Arminio Monforte, Andrea De Luca, Antonio Di Biagio, Enrico Girardi, Nicola Gianotti, Andrea Gori, Giovanni Guaraldi, Giuseppe Lapadula, Miriam Lichtner, Sergio Lo Caputo, Giordano Madeddu, Franco Maggiolo, Giulia Marchetti, Simone Marcotullio, Laura Monno, Cristina Mussini, Massimo Puoti, Eugenia Quiros Roldan, Stefano Rusconi **STATISTICAL AND MONITORING TEAM** A.Cozzi-Lepri, P. Cicconi, I. Fanti, T. Formenti, L. Galli, P. Lorenzini **PARTICIPATING PHYSICIANS AND CENTERS** Italy A. Giacometti, A. Costantini (Ancona); G. Angarano, L. Monno, C. Santoro (Bari); F. Maggiolo, C. Suardi (Bergamo); P. Viale, E. Vanino, G. Verucchi (Bologna); F. Castelli, E. Quiros Roldan, C. Minardi (Brescia); T. Quirino, C. Abeli (Busto Arsizio); P.E. Manconi, P. Piano (Cagliari); J. Vecchiet, K. Falasca (Chieti); L. Sighinolfi, D. Segala (Ferrara); F. Mazzotta, S. Lo Caputo (Firenze); G. Cassola, G. Viscoli, A. Alessandrini, R. Piscopo, G. Mazzarello (Genova); C. Mastroianni, V. Belvisi (Latina); P. Bonfanti, I. Caramma (Lecco); A. P. Castelli (Macerata); M. Galli, A. Lazzarin, G. Rizzardini, M. Puoti, A. d’Arminio Monforte, A.L. Ridolfo, R. Piolini, A. Castagna, S. Salpietro, L. Carenzi, M.C. Moioli, P. Cicconi, G. Marchetti (Milano); C. Mussini, C. Puzzolante (Modena); A. Gori, G. Lapadula (Monza); N. Abrescia, A. Chirianni, M.G. Guida, M. ONOFRIO (Napoli); F. Baldelli, D. Francisci (Perugia); G. Parruti, T. Ursini (Pescara); G. Magnani, M.A. Ursitti (Reggio Emilia); R. Cauda, M. Andreoni, A. Antinori, V. Vullo, A. Cingolani, A. d’Avino, A. Ammassari, L. Gallo, E. Nicastri, R. Acinapura, M. Capozzi, R. Libertone, G. Tebano (Roma); A. Cattelan (Rovigo); M.S. Mura, G. Madeddu (Sassari); P. Caramello, G. Di Perri, G.C. Orofino, S. Bonora, M. Sciandra (Torino); G. Pellizzer, V. Manfrin (Vicenza).

**Nice HIV Cohort** (France):

**Central coordination**: C. Pradier*, E. Fontas, C. Caissotti.

**Participating physicians**: P. Dellamonica, E. Bernard, E. Cua, F. De Salvador-Guillouet, J. Durant, S. Ferrando, V. Mondain-Miton, A. Naqvi, I. Perbost, B. Prouvost-Keller, S. Pillet, P. Pugliese, V. Rahelinirina, P.M. Roger.

**Clinical research assistant**: K. Dollet

**SHCS** (Swiss HIV Cohort Study, Switzerland):

V. Aubert, J. Barth, M. Battegay, E. Bernasconi, J Böni, HC. Bucher, C. Burton-Jeangros, A. Calmy, M. Cavassini, M. Egger, L. Elzi, J. Fehr, J. Fellay, H. Furrer (Chairman of the Clinical and Laboratory Committee), CA. Fux, M. Gorgievski, H. Günthard (President of the SHCS), D. Haerry (deputy of “Positive Council”), B. Hasse, HH. Hirsch, I Hösli, C. Kahlert, L. Kaiser, O. Keiser, T. Klimkait, H. Kovari, B. Ledergerber, G. Martinetti, B. Martinez de Tejada, K. Metzner, N. Müller, D. Nadal, G. Pantaleo, A. Rauch (Chairman of the Scientific Board), A. Regenass, M. Rickenbach (Head of Data Center), C. Rudin (Chairman of the Mother & Child Substudy), P. Schmid, D. Schultze, F. Schöni-Affolter, J. Schüpbach, R. Speck, P. Taffé, P. Tarr, A. Telenti, A. Trkola, P. Vernazza, R. Weber*, S. Yerly.

Financial acknowledgements:

This work was supported by the Highly Active Antiretroviral Therapy Oversight Committee (HAART-OC), a collaborative committee with representation from academic institutions, the European Agency for the Evaluation of Medicinal Products, the United States Food and Drug Administration, the patient community, and all pharmaceutical companies with licensed anti-HIV drugs in the European Union: AbbVie, Boehringer Ingelheim, Bristol-Myers Squibb, Gilead Sciences, ViiV Healthcare, Merck, Pfizer, F.Hoffman-LaRoche and Janssen Pharmaceuticals.

Supported by a grant [grant number CURE/97-46486] from the Health Insurance Fund Council, Amstelveen, the Netherlands, to the AIDS Therapy Evaluation Project Netherlands (ATHENA); by a grant from the Agence Nationale de Recherches sur le SIDA [grant number Action Coordonnée no.7, Cohortes], to the Aquitaine Cohort; The Australian HIV Observational Database (AHOD) is funded as part of the Asia Pacific HIV Observational Database, a program of The Foundation for AIDS Research, amfAR, and is supported in part by a grant from the U.S. National Institutes of Health’s National Institute of Allergy and Infectious Diseases (NIAID) (Grant No. U01-AI069907) and by unconditional grants from Merck Sharp & Dohme; Gilead Sciences; Bristol-Myers Squibb; Boehringer Ingelheim; Roche; Pfizer; GlaxoSmithKline; Janssen Pharmaceuticals. The Kirby Institute is funded by The Australian Government Department of Health and Ageing, and is affiliated with the Faculty of Medicine, The University of New South Wales. By grants from the Fondo de Investigación Sanitaria [grant number FIS 99/0887] and Fundación para la Investigación y la Prevención del SIDA en Espanã [grant number FIPSE 3171/00], to the Barcelona Antiretroviral Surveillance Study (BASS); by the National Institute of Allergy and Infectious Diseases, National Institutes of Health [grants number 5U01AI042170-10, 5U01AI046362-03], to the Terry Beirn Community Programs for Clinical Research on AIDS (CPCRA); by grants from the BIOMED 1 [grant number CT94-1637] and BIOMED 2 [grant number CT97-2713] programs and the fifth framework program [grant number QLK2-2000-00773] of the European Commission and grants from Bristol-Myers Squibb, GlaxoSmithKline, Boehringer Ingelheim, and Roche, to the EuroSIDA study; by unrestricted educational grants of AbbVie, Bristol-Myers Squibb, Gilead Sciences, GlaxoSmithKline, Pfizer, Janssen Pharmaceuticals to the Italian Cohort Naive to Antiretrovirals (The ICONA Foundation); and by a grant from the Swiss National Science Foundation, to the Swiss HIV Cohort Study (SHCS).

## References

[B1] Wyatt CM Klotman PE Antiretroviral therapy and the kidney: balancing benefit and risk in patients with HIV infection Expert Opin Drug Saf 2006 5 275 287 10.1517/14740338.5.2.275 16503748

[B2] Ryom L Mocroft A Kirk O Worm SW Kamara DA Reiss P Ross M Fux CA Morlat P Moranne O Smith C Lundgren JD Association between antiretroviral exposure and renal impairment among HIV-positive persons with normal baseline renal function: the D:A:D study J Infect Dis 2013 207 1359 1369 10.1093/infdis/jit043 23382571 PMC3610424

[B3] Phair J Palella F Renal disease in HIV-infected individuals Curr Opin HIV AIDS 2011 6 285 289 10.1097/COH.0b013e3283476bc3 21519246 PMC3266688

[B4] Mocroft A Kirk O Reiss P De WS Sedlacek D Beniowski M Gatell J Phillips AN Ledergerber B Lundgren JD Estimated glomerular filtration rate, chronic kidney disease and antiretroviral drug use in HIV-positive patients AIDS 2010 24 1667 1678 10.1097/QAD.0b013e328339fe53 20523203

[B5] Flandre P Pugliese P Cuzin L Bagnis CI Tack I Cabie A Poizot-Martin I Katlama C Brunet-Francois C Yazdanpanah Y Dellamonica P Risk factors of chronic kidney disease in HIV-infected patients Clin J Am Soc Nephrol 2011 6 1700 1707 10.2215/CJN.09191010 21566114

[B6] Levey AS Atkins R Coresh J Cohen EP Collins AJ Eckardt KU Nahas ME Jaber BL Jadoul M Levin A Powe NR Rossert J Wheeler DC Lameire N Eknoyan G National Kidney Foundation practice guidelines for chronic kidney disease: evaluation, classification, and stratification Ann Intern Med 2003 139 137 147 10.7326/0003-4819-139-2-200307150-00013 12859163

[B7] Levey AS Eckardt KU Tsukamoto Y Levin A Coresh J Rossert J De ZD Hostetter TH Lameire N Eknoyan G Definition and classification of chronic kidney disease: a position statement from Kidney Disease: Improving Global Outcomes (KDIGO) Kidney Int 2005 67 2089 2100 10.1111/j.1523-1755.2005.00365.x 15882252

[B8] Eckardt KU Berns JS Rocco MV Kasiske BL Definition and classification of CKD: the debate should be about patient prognosis–a position statement from KDOQI and KDIGO Am J Kidney Dis 2009 53 915 920 10.1053/j.ajkd.2009.04.001 19406541

[B9] Hunsicker LG Adler S Caggiula A England BK Greene T Kusek JW Rogers NL Teschan PE Predictors of the progression of renal disease in the Modification of Diet in Renal Disease Study Kidney Int 1997 51 1908 1919 10.1038/ki.1997.260 9186882

[B10] Eriksen BO Tomtum J Ingebretsen OC Predictors of declining glomerular filtration rate in a population-based chronic kidney disease cohort Nephron Clin Pract 2010 115 c41 c50 20173349 10.1159/000286349

[B11] Eriksen BO Ingebretsen OC The progression of chronic kidney disease: a 10-year population-based study of the effects of gender and age Kidney Int 2006 69 375 382 10.1038/sj.ki.5000058 16408129

[B12] Stevens PE Levin A Evaluation and management of chronic kidney disease: synopsis of the kidney disease: improving global outcomes 2012 clinical practice guideline Ann Intern Med 2013 158 825 830 10.7326/0003-4819-158-11-201306040-00007 23732715

[B13] Keller C Katz R Sarnak MJ Fried LF Kestenbaum B Cushman M Shlipak MG Inflammatory biomarkers and decline in kidney function in the elderly: the Cardiovascular Health Study Nephrol Dial Transplant 2010 25 119 124 10.1093/ndt/gfp429 19734138 PMC2910326

[B14] Kop WJ Seliger SL Fink JC Katz R Odden MC Fried LF Rifkin DE Sarnak MJ Gottdiener JS Longitudinal association of depressive symptoms with rapid kidney function decline and adverse clinical renal disease outcomes Clin J Am Soc Nephrol 2011 6 834 844 10.2215/CJN.03840510 21393483 PMC3069377

[B15] Lin J Fung TT Hu FB Curhan GC Association of dietary patterns with albuminuria and kidney function decline in older white women: a subgroup analysis from the Nurses’ Health Study Am J Kidney Dis 2011 57 245 254 10.1053/j.ajkd.2010.09.027 21251540 PMC3026604

[B16] Longenecker CT Scherzer R Bacchetti P Lewis CE Grunfeld C Shlipak MG HIV viremia and changes in kidney function AIDS 2009 23 1089 1096 10.1097/QAD.0b013e32832a3f24 19352136 PMC3725756

[B17] Rifkin DE Shlipak MG Katz R Fried LF Siscovick D Chonchol M Newman AB Sarnak MJ Rapid kidney function decline and mortality risk in older adults Arch Intern Med 2008 168 2212 2218 10.1001/archinte.168.20.2212 19001197 PMC2879064

[B18] Scherzer R Estrella M Li Y Choi AI Deeks SG Grunfeld C Shlipak MG Association of tenofovir exposure with kidney disease risk in HIV infection AIDS 2012 26 867 875 10.1097/QAD.0b013e328351f68f 22313955 PMC3736566

[B19] Alves TP Hulgan T Wu P Sterling TR Stinnette SE Rebeiro PF Vincz AJ Bruce M Ikizler TA Race, kidney disease progression, and mortality risk in HIV-infected persons Clin J Am Soc Nephrol 2010 5 2269 2275 10.2215/CJN.00520110 20876679 PMC2994089

[B20] Cao Y Gong M Han Y Xie J Li X Zhang L Li Y Song X Zhu T Li T Prevalence and risk factors for chronic kidney disease among HIV-infected ART-naive patients in Mainland China: a multicenter cross-sectional study Nephrology (Carlton) 2013 3 3 10.1111/nep.12031 23311442

[B21] Lucas GM Lau B Atta MG Fine DM Keruly J Moore RD Chronic kidney disease incidence, and progression to end-stage renal disease, in HIV-infected individuals: a tale of two races J Infect Dis 2008 197 1548 1557 10.1086/587994 18422458 PMC2553209

[B22] Friis-Moller N Sabin CA Weber R D’Arminio MA El-Sadr WM Reiss P Thiebaut R Morfeldt L De WS Pradier C Calvo G Law MG Kirk O Phillips AN Lundgren JD Combination antiretroviral therapy and the risk of myocardial infarction N Engl J Med 2003 349 1993 2003 14627784 10.1056/NEJMoa030218

[B23] Moger V Kumar SK Sakhuja V Joshi K Walker R Kohli HS Sud K Gupta KL Jha V Rapidly progressive renal failure in type 2 diabetes in the tropical environment: a clinico-pathological study Ren Fail 2005 27 595 600 10.1080/08860220500200205 16152999

[B24] Crum-Cianflone N Ganesan A Teneza-Mora N Riddle M Medina S Barahona I Brodine S Prevalence and factors associated with renal dysfunction among HIV-infected patients AIDS Patient Care STDS 2010 24 353 360 10.1089/apc.2009.0326 20515419 PMC2933561

[B25] Dauchy FA Lawson-Ayayi S de La FR Bonnet F Rigothier C Mehsen N Miremont-Salame G Cazanave C Greib C Dabis F Dupon M Increased risk of abnormal proximal renal tubular function with HIV infection and antiretroviral therapy Kidney Int 2011 80 302 309 10.1038/ki.2011.124 21544066

[B26] Deti EK Thiebaut R Bonnet F Lawson-Ayayi S Dupon M Neau D Pellegrin JL Malvy D Tchamgoue S Dabis F Morlat P Prevalence and factors associated with renal impairment in HIV-infected patients, ANRS C03 Aquitaine Cohort, France HIV Med 2010 11 308 317 10.1111/j.1468-1293.2009.00780.x 20002500

[B27] Gallant JE Parish MA Keruly JC Moore RD Changes in renal function associated with tenofovir disoproxil fumarate treatment, compared with nucleoside reverse-transcriptase inhibitor treatment Clin Infect Dis 2005 40 1194 1198 10.1086/428840 15791522

[B28] Fernando SK Finkelstein FO Moore BA Weissman S Prevalence of chronic kidney disease in an urban HIV infected population Am J Med Sci 2008 335 89 94 10.1097/MAJ.0b013e31812e6b34 18277114

[B29] Hanratty R Chonchol M Miriam DL Beaty BL Estacio RO Mackenzie TD Hurley LP Linas SL Steiner JF Havranek EP Incident chronic kidney disease and the rate of kidney function decline in individuals with hypertension Nephrol Dial Transplant 2010 25 801 807 10.1093/ndt/gfp534 19889870 PMC2828608

[B30] Post FA Wyatt CM Mocroft A Biomarkers of impaired renal function Curr Opin HIV AIDS 2010 5 524 530 10.1097/COH.0b013e32833f203e 20978396

[B31] Wyatt CM Winston JA Malvestutto CD Fishbein DA Barash I Cohen AJ Klotman ME Klotman PE Chronic kidney disease in HIV infection: an urban epidemic AIDS 2007 21 2101 2103 10.1097/QAD.0b013e3282ef1bb4 17885301

[B32] Eftimovska N Stojceva-Taneva O Polenakovic M Slow progression of chronic kidney disease and what it is associated with Prilozi 2008 29 153 165 18709007

[B33] Seliger SL Davis C Stehman-Breen C Gender and the progression of renal disease Curr Opin Nephrol Hypertens 2001 10 219 225 10.1097/00041552-200103000-00010 11224697

[B34] Turin TC Coresh J Tonelli M Stevens PE de Jong PE Farmer CK Matsushita K Hemmelgarn BR One-year change in kidney function is associated with an increased mortality risk Am J Nephrol 2012 36 41 49 10.1159/000339289 22699706

[B35] Levey AS Bosch JP Lewis JB Greene T Rogers N Roth D A more accurate method to estimate glomerular filtration rate from serum creatinine: a new prediction equation. Modification of Diet in Renal Disease Study Group Ann Intern Med 1999 130 461 470 10.7326/0003-4819-130-6-199903160-00002 10075613

[B36] Levey AS Stevens LA Schmid CH Zhang YL Castro AF III Feldman HI Kusek JW Eggers P Van LF Greene T Coresh J A new equation to estimate glomerular filtration rate Ann Intern Med 2009 150 604 612 10.7326/0003-4819-150-9-200905050-00006 19414839 PMC2763564

[B37] Ibrahim F Hamzah L Jones R Nitsch D Sabin C Post FA Comparison of CKD-EPI and MDRD to estimate baseline renal function in HIV-positive patients Nephrol Dial Transplant 2012 27 2291 2297 10.1093/ndt/gfr657 22121232

[B38] Vrouenraets SM Fux CA Wit FW Garcia EF Brinkman K Hoek FJ van Straalen JP Furrer H Krediet RT Reiss P A comparison of measured and estimated glomerular filtration rate in successfully treated HIV-patients with preserved renal function Clin Nephrol 2012 77 311 320 10.5414/CN107214 22445475

[B39] Soliman AR Fathy A Khashab S Shaheen N Comparison of abbreviated modification of diet in renal disease formula (aMDRD) and the Cockroft-Gault adjusted for body surface (aCG) equations in stable renal transplant patients and living kidney donors Ren Fail 2013 35 94 97 10.3109/0886022X.2012.731970 23181772

[B40] Zamora E Lupon J Vila J Urrutia A de Antonio M Sanz H Grau M Ara J Bayés-Genís A Estimated glomerular filtration rate and prognosis in heart failure: value of the Modification of Diet in Renal Disease Study-4, chronic kidney disease epidemiology collaboration, and cockroft-gault formulas J Am Coll Cardiol 2012 59 1709 1715 10.1016/j.jacc.2011.11.066 22554602

[B41] Delanaye P Radermecker RP Rorive M Depas G Krzesinski JM Indexing glomerular filtration rate for body surface area in obese patients is misleading: concept and example Nephrol Dial Transplant 2005 20 2024 2028 10.1093/ndt/gfh983 16030047

[B42] Delanaye P Mariat C Cavalier E Krzesinski JM Errors induced by indexing glomerular filtration rate for body surface area: reductio ad absurdum Nephrol Dial Transplant 2009 24 3593 3596 10.1093/ndt/gfp431 19734136

[B43] Hosmer DW Lemeshow S Applied Logistic Regression 2000 New York: John Wiley & Sons, Inc Ref Type: Edited Book

