# Supplementary material for: Development of a definition for Rapid Progression (RP) of renal function in HIV-positive persons: the D:A:D study
Source: BMC Nephrol. 2014 Mar 25;15:51. doi: 10.1186/1471-2369-15-51 (PMC3987148; doi:10.1186/1471-2369-15-51)
Supplement: Additional file 1: Table S1 — aBaseline characteristics of patients included in analyses of rapid progression, according to availability of data. [file 1471-2369-15-51-S1.docx]

Additional file 1: Table S1: ^a^Baseline characteristics of patients included in analyses of rapid progression, according to availability of data

|  |  | Patients with eGFR data available | >2 years follow-up | | |
| --- | --- | --- | --- | --- | --- |
|  |  |  | Unrestricted* | ≥3 eGFR per year | ≥2 eGFR per year |
| Eligible patients | Number  (%) | 22603  (100) | 19309  (85.4) | 10756  (47.6) | 17211  (76.1) |
| Average eGFR | Median (IQR) | 109  (99-123) | 109  (99-123) | 109  (99-122) | 109  (99-122) |
| eGFR >120 ml/min | Number (%) | 6613 (29.3) | 5589 (29.0) | 3015 (28.0) | 4910 (28.5) |
| Gender | Male | 16438 (72.7) | 13980 (72.4) | 7825 (72.8) | 12506 (72.7) |
| Race | White | 10573 (46.8) | 9017 (46.7) | 4337 (40.3) | 7902 (45.9) |
|  | Black | 1806 (8.0) | 1577 (8.2) | 921 (8.6) | 1421 (8.3) |
|  | Other | 510 (2.3) | 446 (2.3) | 222 (2.1) | 404 (2.4) |
|  | Unknown | 9714 (43.0) | 8269 (42.8) | 5276 (49.1) | 7484 (43.5) |
| Age group | >50 years | 2238 (9.9) | 1943 (10.1) | 1173 (10.9) | 1761 (10.2) |
| ^b^Hypertension | Yes | 2519 (11.1) | 2202 (11.4) | 1283 (11.9) | 1986 (11.5) |
| ^c^Diabetes | Yes | 659 (2.9) | 587 (3.0) | 360 (3.4) | 541 (3.1) |
| Mode of HIV acquisition | Homosexual | 10006 (44.3) | 8524 (44.2) | 4920 (45.7) | 7705 (44.8) |
|  | IDU | 3058 (13.5) | 2574 (13.3) | 1255 (11.7) | 2195 (12.8) |
|  | Heterosexual | 8095 (35.8) | 6988 (36.2) | 3903 (36.3) | 6221 (36.2) |
|  | Other | 1444 (6.4) | 1223 (6.3) | 678 (6.3) | 1090 (6.3) |
| BMI at study entry (kg/m^2^) | <18 | 633 (2.8) | 534 (2.8) | 298 (2.8) | 478 (2.8) |
|  | ≥18, <26 | 15865 (70.2) | 13630 (70.6) | 7540 (70.1) | 12171 (70.7) |
|  | ≥26, <30 | 3716 (16.4) | 3233 (16.7) | 1769 (16.5) | 2885 (16.8) |
|  | ≥30 | 1423 (6.3) | 1233 (6.4) | 691 (6.4) | 1065 (6.2) |
|  | Unknown | 966 (4.3) | 679 (3.5) | 458 (4.3) | 612 (3.6) |
| ^d^Hepatitis C positive | Yes | 2765 (12.2) | 2305 (11.9) | 1139 (10.6) | 1979 (11.5) |
| ^e^Hepatitis B positive | Yes | 2773 (12.3) | 2358 (12.2) | 1365 (12.7) | 2099 (12.2) |
| Smoking status | Current | 9544 (42.2) | 8210 (42.5) | 4353 (40.5) | 7224 (42.0) |
|  | Previous | 3930 (17.4) | 3429 (17.8) | 1968 (18.3) | 3112 (18.1) |
|  | Never | 5824 (25.8) | 5093 (26.4) | 2817 (26.2) | 4572 (26.6) |
|  | Unknown | 3305 (14.6) | 2577 (13.4) | 1618 (15.0) | 2303 (13.4) |
| Current CD4 count (cells/mm^3^) | <200 | 2823 (12.5) | 2317 (12.0) | 1430 (13.3) | 2084 (12.1) |
|  | 200-349 | 4912 (21.7) | 4158 (21.5) | 2433 (22.6) | 3743 (21.8) |
|  | 350-499 | 5565 (24.6) | 4768 (24.7) | 2686 (25.0) | 4268 (24.8) |
|  | >500 | 9145 (40.5) | 7955 (41.2) | 4136 (38.5) | 7014 (40.8) |
|  | Missing | 158 (0.7) | 111 (0.6) | 71 (0.7) | 102 (0.6) |

Abbreviations: eGFR, estimated glomerular filtration rate; IDU, injecting drug user;

a Baseline was defined as the defined as the time, during prospective follow-up, of the first eGFR measurement after 1 January 2004.

b Hypertension was defined as a blood pressure of >150/>100 mmHg or use of antihypertensive drugs

c Diabetes was defined as receipt of antidiabetic treatment or verification of diabetes in a case report form

d Hepatitis C was defined as detection of antibody to HCV plus detection or unknown presence of HCV RNA.

e Hepatitis B was defined as detection of HBV surface antigen, detection of HBV e antigen, or detection of HBV DNA plus antibody to HBV e antigen.

* Unrestricted was defined as no restriction concerning the number of eGFR measurements obtained per year
