# Supplementary material for: Development of a definition for Rapid Progression (RP) of renal function in HIV-positive persons: the D:A:D study
Source: BMC Nephrol. 2014 Mar 25;15:51. doi: 10.1186/1471-2369-15-51 (PMC3987148; doi:10.1186/1471-2369-15-51)
Supplement: Additional file 2: Table S2 — Percentage who progressed to incident CKD or death according to different definitions of RP. [file 1471-2369-15-51-S2.docx]

Additional file 2: Table S2: Percentage who progressed to incident CKD or death according to different definitions of RP

| Years of follow-up considered | Number of eGFR per year | Excluded in analysis | RP definition A | | | | RP definition B | | | | | | |
| --- | --- | --- | --- | --- | --- | --- | --- | --- | --- | --- | --- | --- | --- |
|  |  |  | ^a^RP (PPV) | ^b^non-RP  (1-NPV) | Sensitivity  (%) | Specificity  (%) | RP (PPV) | | non-RP  (1-NPV) | | Sensitivity  (%) | | Specificity  (%) |
|  |  |  | Declines of >5 ml/min/1.73m^2^ required to meet the RP definition | | | | | | | | | | |
| Four | Three | 768/18948 (4.1) | 16/108(14.8) | 64/3547 (1.8) | 20.0 | 97.4 | 0/15 (0.0) | | 80/3640 (2.2) | | 0.0 | | 99.6 |
| Four | Two | 681/14305 (4.8) | 30/256 (11.7) | 137/8042 (1.7) | 18.0 | 97.2 | 3/43 (7.0) | | 164/8255 (2.0) | | 1.8 | | 99.5 |
| Three | Three | 684/16228 (4.2) | 19/195 (9.7) | 145/6180 (2.3) | 11.6 | 97.2 | 6/85 (7.1) | | 158/6290 (2.5) | | 3.7 | | 98.7 |
| Three | Two | 557/10320 (5.0) | 27/369 (7.3) | 264/11914 (2.2) | 9.3 | 97.1 | 9/173 (5.2) | | 282/12110 (2.3) | | 3.1 | | 98.6 |
| Two | Three | 535/11847 (4.5) | 23/355 (6.5) | 290/10401 (2.8) | 7.3 | 96.8 | 27/476 (5.7) | | 286/10280 (2.8) | | 8.6 | | 95.7 |
| Two | Two | 366/5392 (6.8) | 38/602 (6.3) | 444/16609 (2.7) | 7.9 | 96.6 | 45/779 (5.8) | | 437/16432 (2.7) | | 9.3 | | 95.6 |
|  |  |  | Declines of >3 ml/min/1.73m^2^ required to meet the RP definition | | | | | | | | | | |
| Four | Three | 768/18948 (4.1) | 22/218 (10.1) | 58/3437 (1.7) | 27.5 | 94.5 | 3/37 (8.1) | 77/3618 (1.7) | | 3.8 | | 99.0 | |
| Four | Two | 681/14305 (4.8) | 38/506 (7.5) | 128/7792 (1.7) | 22.8 | 94.2 | 7/97 (7.2) | 160/8201 (1.7) | | 4.2 | | 98.9 | |
| Three | Three | 684/16228 (4.2) | 26/325 (8.0) | 138/6050 (2.3) | 15.9 | 95.2 | 11/151 (7.3) | 153/6224 (2.3) | | 6.7 | | 97.7 | |
| Three | Two | 557/10320 (5.0) | 35/602 (5.8) | 256/11681 (2.2) | 12.0 | 95.3 | 17/292 (5.8) | 274/11991 (2.2) | | 5.8 | | 97.7 | |
| Two | Three | 535/11847 (4.5) | 32/527 (6.1) | 281/10229 (2.7) | 10.2 | 95.3 | 37/633 (5.8) | 276/10123 (2.7) | | 11.8 | | 94.3 | |
| Two | Two | 366/5392 (6.8) | 56/885 (6.3) | 426/16326 (2.6) | 11.6 | 95.0 | 61/1021(6.0) | 421/16190 (2.6) | | 12.7 | | 94.3 | |

^a^RP: Rapid Progressors who developed CKD (i.e., the positive predictive value (PPV))

^b^Non-RP: non-Rapid Progressors who developed CKD (i.e., 1-negative predictive value (NPV))

*two consecutive eGFR measurements <60 ml/min/1.73m^2^
